# Cytomegalovirus-associated ulceration of gastric conduit after chemoradiotherapy following esophagectomy for cancer

**DOI:** 10.1007/s10388-014-0441-9

**Published:** 2014-06-06

**Authors:** Yasunori Matsuda, Satoru Kishida, Hikaru Miyamoto, Shigeru Lee, Masato Okawa, Yushi Fujiwara, Ryoya Hashiba, Eijiro Edagawa, Sayaka Tanaka, Masahiko Osawa, Harushi Osugi

**Affiliations:** 1 Department of Gastroenterological Surgery, Graduate School of Medicine, Osaka City University, 1-4-3 Asahimachi, Abeno-ku, Osaka, 545-8585 Japan; 2 Department of Diagnostic Pathology, Graduate School of Medicine, Osaka City University, Osaka, Japan

**Keywords:** Cytomegalovirus, Gastric conduit, Radical esophagectomy, Ulceration

## Abstract

A 64-year-old man underwent radical esophagectomy for cancer and simultaneous reconstruction using the gastric conduit through the posterior mediastinum. Two courses of adjuvant chemotherapy were performed. Twenty-eight months postoperatively, recurrence of the cancer was detected in the mediastinal lymph nodes, and he underwent concurrent chemoradiotherapy and boost chemotherapy. Endoscopy was then performed to investigate the cause of epigastralgia, and multiple ulcerations were found in the lesser curvature of the gastric conduit. Although a proton-pump inhibitor was orally administered, the ulceration was intractable. Re-examination of the original biopsy specimens and serological testing revealed positivity for cytomegalovirus. The ulcers began to heal after administration of foscarnet sodium. After the treatment, no signs of exacerbation associated with reinstitution of chemotherapy were observed.

## Introduction

The spectrum of human illness caused by cytomegalovirus (CMV) is diverse and mostly dependent on the host. CMV generally produces an asymptomatic or minimally symptomatic acute illness such as mononucleosis in immunocompetent hosts [1]. In immunocompromised hosts, CMV infection can result in a broad array of clinical manifestations, including retinitis, pneumonitis, encephalitis, hepatitis, and gastrointestinal tract ulceration [2, 3].

CMV-associated disease can involve any part of the gastrointestinal tract. However, the most common locations include the esophagus and colon. Symptom presentation depends on the anatomic location of the infection. A correct diagnosis is vital for an appropriate treatment.

We herein present a rare case involving CMV-associated ulceration of the gastric conduit in a patient who underwent postoperative multimodal therapy for lymph node recurrence of esophageal cancer.

## Case report

**Fig. 1:**
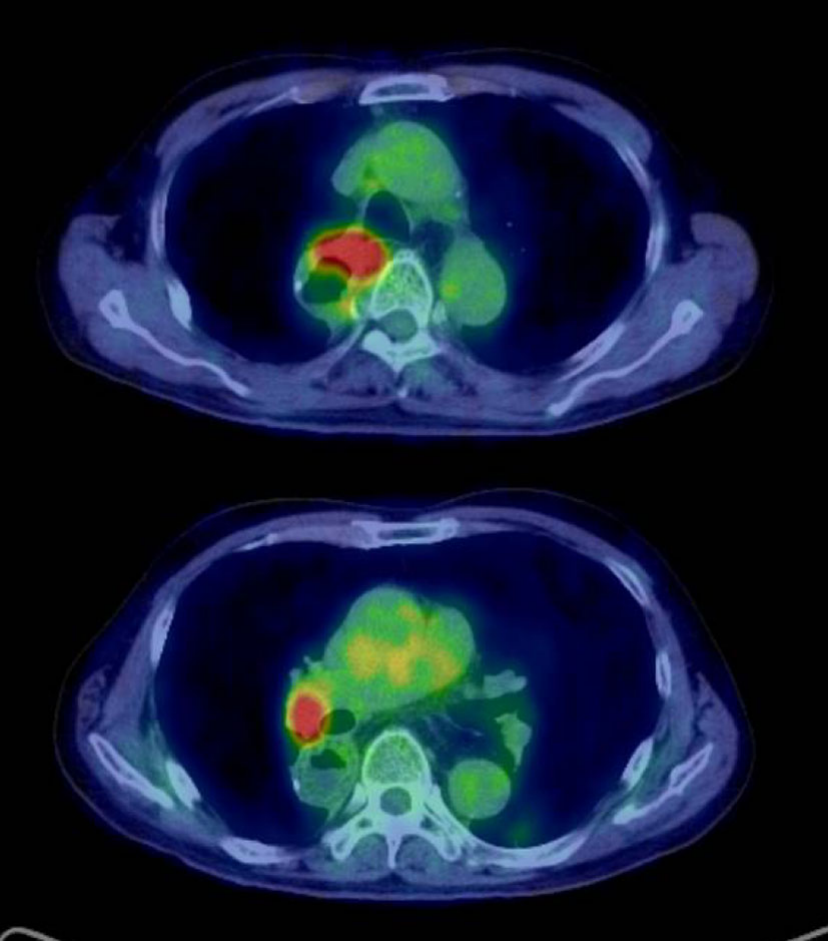
18-fluorodeoxyglucose positron emission tomography revealed abnormal accumulation in the mediastinal lymph nodes

**Fig. 2:**
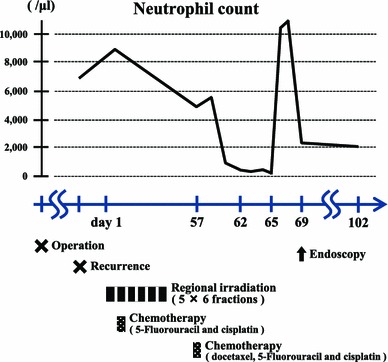
Change in neutrophil count during postoperative multimodal therapy for esophageal cancer recurrence

**Fig. 3:**
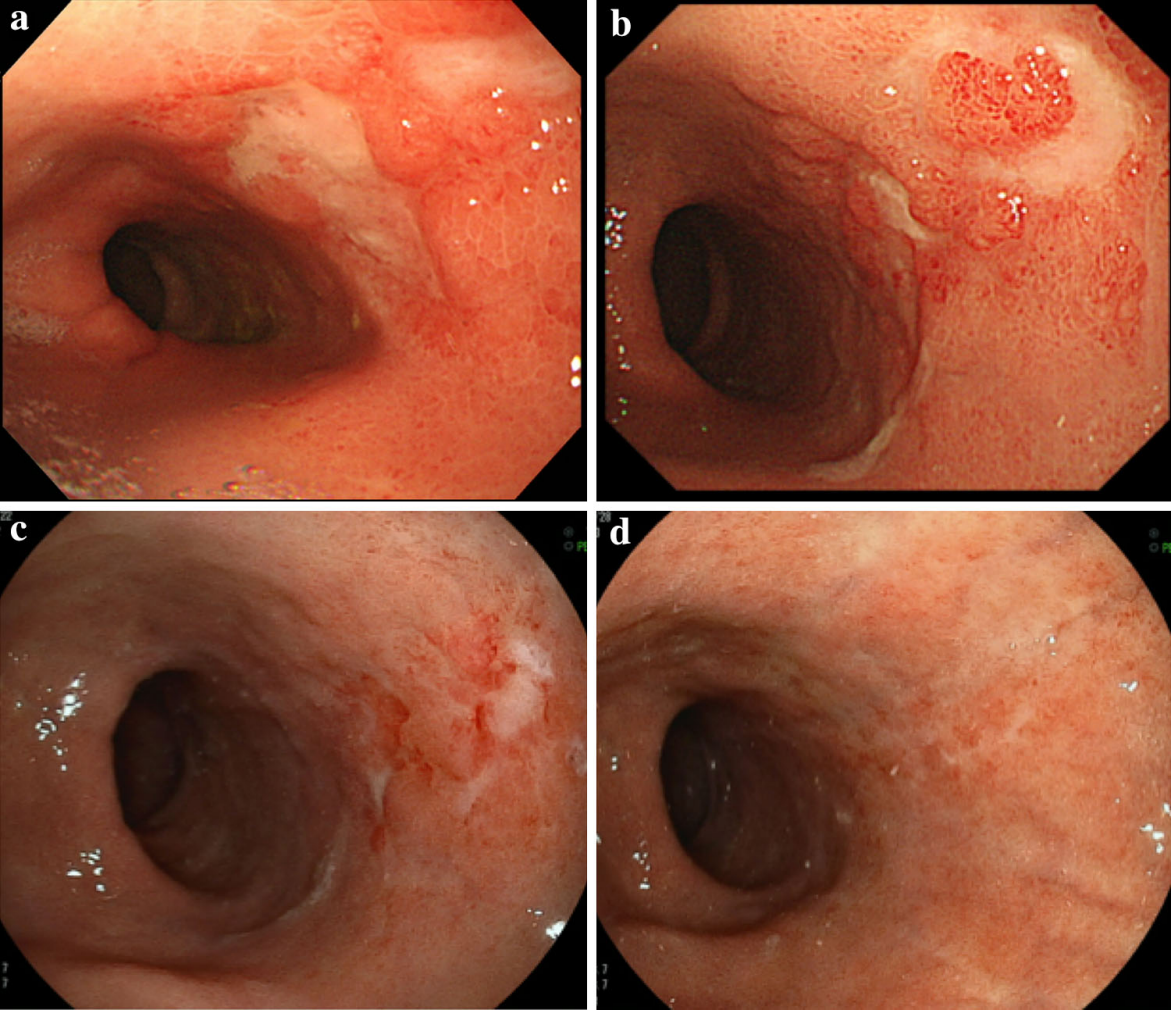
Endoscopic photographs of transition of the ulceration in the lesser curvature of the gastric conduit. **a** After completion of chemotherapy. **b** After 1 month of oral administration of a proton-pump inhibitor. **c** After 2 weeks of administration of foscarnet sodium. **d** After reinstitution of chemotherapy

**Fig. 4:**
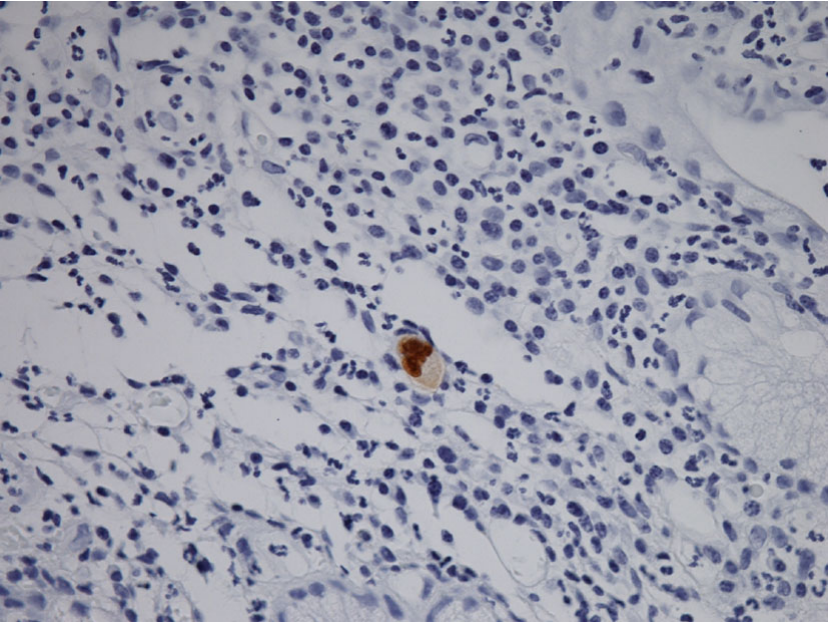
Representative immunohistochemical staining of biopsy specimens. Intranuclear inclusions were positively stained with anti-cytomegalovirus antibodies

A 64-year-old man underwent radical esophagectomy for cancer and simultaneous reconstruction using the gastric conduit through the posterior mediastinum. The pathological diagnosis of the esophageal cancer was moderately differentiated squamous cell carcinoma, and the TNM classification (7th edition) was T3N1M0 (stage IIIA). According to the protocol described in a Japan Clinical Oncology Group study [4], two courses of adjuvant chemotherapy were performed (5-fluorouracil, 800 mg/m^2^, days 1–5; cisplatin, 80 mg/m^2^, day 1). Twenty-eight months after the surgery, 18-fluorodeoxyglucose positron emission tomography revealed that the tumor had recurred in the mediastinal lymph nodes (Fig. 1). The patient then underwent concurrent chemoradiotherapy (regional irradiation, total dose of 60 Gy in 30 fractions; 5-fluorouracil, 700 mg/m^2^, days 7–11; cisplatin, 70 mg/m^2^, day 7), and boost chemotherapy (docetaxel, 56 mg/m^2^, day 56; cisplatin, 60 mg/m^2^, day 56; 5-fluorouracil, 560 mg/m^2^, days 56–60). After completion of the chemotherapy, neutropenia of National Cancer Institute grade 3 and epigastralgia developed (Fig. 2). Endoscopy revealed multiple ulcerations in the lesser curvature of the gastric conduit (Fig. 3a). Although a proton-pump inhibitor (lansoprazole, 30 mg/day) was orally administered for 1 month, the ulceration was intractable (Fig. 3b). Re-evaluation of the biopsy specimen obtained at the time of the first endoscopy revealed intranuclear inclusions positively stained with anti-CMV antibodies (Fig. 4). Serological testing revealed positivity for CMV immunoglobulin G antibody, negativity for immunoglobulin M antibody, and the presence of CMV pp65 antigenemia. Scheduled boost chemotherapy was deferred, and foscarnet sodium was administered at 180 mg/kg/day for 2 weeks. After the treatment, upper endoscopy revealed evidence of healing of the ulcerations (Fig. 3c) and no signs of exacerbation associated with reinstitution of chemotherapy (Fig. 3d). Although the patient completed six courses of palliative chemotherapy (docetaxel, 40–60 mg/m^2^), lung and liver metastases developed and he died 51 months after the surgery.

## Discussion

The two main clinical scenarios in which CMV acts as an opportunistic pathogen causing gastrointestinal manifestations in immunocompromised hosts are autoimmune deficiency syndrome (AIDS) and transplantation. Other causes of depressed immunity, such as iatrogenic causes, can also result in CMV infections in the gastrointestinal tract [5]. Like other members of the herpesvirus family, CMV establishes latent infection after the resolution of acute infection [6]. T cells play an important role in controlling viral replication and disease, but cannot completely eliminate the virus. High frequencies of specific CMV CD4+ and CD8+ T cell responses have been demonstrated in immunocompetent hosts [7]. Reactivation of CMV may occur at any time during the life of the human host, and the risk is higher in the setting of systemic immunosuppression. In the present case, the CMV infection appeared to have caused ulceration due to depression of cellular immunity after postoperative multimodal therapy for esophageal cancer recurrence.

CMV infection is a rare cause of intractable gastric ulceration [5]. CMV has been associated with gastric ulceration in immunocompromised hosts, in whom it can cause multiple large, shallow ulcerations [5, 8, 9]. Although CMV-associated ulceration of a gastric conduit used for a reconstruction procedure has never been reported, the present case showed typical imaging findings. Thus, we suspect that the intractable ulcerations were caused by CMV infection due to depression of immunity.

The diagnosis of CMV-associated ulceration was confirmed with histopathology in the present case. Histologic examination of tissue biopsies is useful for the diagnosis of invasive CMV infection. For gastrointestinal disease, plasma or whole blood CMV DNA load tests are sometimes negative, so the diagnosis relies upon histopathology of a tissue biopsy specimen. Diagnosis is based on the presence of inclusion bodies, typically basophilic intranuclear inclusions, although eosinophilic cytoplasmic inclusions may also be seen; the diagnosis of CMV in tissue sections can be confirmed with immunohistochemical staining [3]. Antibodies to CMV antigens for use in immunohistology are widely available and may be used on both frozen and formalin-fixed material [10].

Currently, several antiviral agents are available for systemic treatment of CMV infection, including ganciclovir, valganciclovir, foscarnet, and cidofovir. Foscarnet is a pyrophosphate analog that inhibits viral replication by selective binding to viral DNA polymerase [11] and is principally used for the treatment of ganciclovir-resistant CMV infections in patients with AIDS or who have undergone transplantation. However, ganciclovir and valganciclovir are both associated with bone marrow suppression and should be used with caution or substituted with other antiviral agents in patients with pre-existing cytopenias [12]. In the present case, it was necessary to avoid bone marrow suppression as an adverse effect associated with the antiviral agent because the patient required additional chemotherapy for the lymph node relapse. We successfully treated CMV-associated ulceration using foscarnet without any adverse effects and reinstituted chemotherapy for the recurrence of esophageal cancer.

In patients with AIDS and transplantation, primary prophylaxis with an antiviral agent is effective [13, 14]. However, decisions regarding prophylaxis for patients undergoing chemotherapy or radiation therapy must take the lack of clinical evidence into consideration. The risk of exteriorization of CMV is significantly reduced by the preemptive administration of an antiviral agent in patients with CMV infection [15]. Serological testing including monoclonal antibody, quantitative PCR, and pp65 antigenemia assays may be the most practical tools for preemptive CMV therapy. Patient education about the potential symptoms and signs of CMV infection in the gastrointestinal tract is also thought to be effective.

This case report provides information applicable to practical physicians who treat patients with immunosuppression of various causes. Although rare, CMV infection must be considered when immunosuppressed patients develop gastrointestinal tract ulceration.
